# Characterization of novel carcinoma cell lines for the analysis of therapeutical strategies fighting pancreatic cancer

**DOI:** 10.1186/s13578-015-0038-6

**Published:** 2015-08-28

**Authors:** Dietmar Zechner, Florian Bürtin, Jonas Amme, Tobias Lindner, Tobias Radecke, Stefan Hadlich, Jens-Peter Kühn, Brigitte Vollmar

**Affiliations:** Institute for Experimental Surgery, Rostock University Medical Center, University of Rostock, Schillingallee 69a, 18057 Rostock, Germany; Core Facility Small Animal Imaging, Rostock University Medical Center, Schillingallee 69a, 18057, Rostock, Germany; Department of Diagnostic Radiology and Neuroradiology, University Medicine Greifswald, Sauerbruchstr. 1, 17489 Greifswald, Germany

**Keywords:** Syngeneic cancer model, Pancreatic ductal adenocarcinoma, Noninvasive imaging, Cancer remission, Combinatorial therapy, Cancer immunology, Desmoplasia

## Abstract

**Background:**

Preclinical evaluations of chemotherapies depend on clinically relevant animal models for pancreatic cancer. The injection of syngeneic murine adenocarcinoma cells is one efficient option to generate carcinomas in mice with an intact immune system. However, this option is constrained by the paucity of appropriate cell lines.

**Results:**

The murine pancreatic adenocarcinoma cell lines 6606PDA and 7265PDA were compared to the 6606l cell line isolated from a liver metastasis from mice suffering from pancreatic cancer. In tissue culture 6606PDA and 6606l proliferated faster than 7265PDA. 7265PDA cells were, however, significantly more sensitive to gemcitabine as assessed by BrdU-incorporation and trypan blue exclusion assays in vitro. Within 1 week after injection of either one of these three cell lines into the pancreas of C57BL/6J mice, carcinomas were observed by T2 weighted magnetic resonance imaging and histology. Three weeks after injecting 6606PDA or 6606l cells large carcinomas could be characterized, which were surrounded by extensive desmoplastic reaction. After injection of 7265PDA cells, however, remission of cancer was observed between the first and the third week. Compared to 6606PDA cell derived carcinomas a higher apparent diffusion coefficient was quantified by diffusion weighted magnetic resonance imaging in these tumors. This correlated with reduced cancer cell density observed on histological sections.

**Conclusion:**

All three cell lines can be used in vitro for testing combinatorial therapies with gemcitabine. The 6606PDA and 6606l cell lines but not the 7265PDA cell line can be used for evaluating distinct therapies in a syngeneic carcinoma model using C57BL/6J mice. Diffusion-weighted MRI proved to be an appropriate method to predict tumor remission.

## Background

Although multiple radiation therapies and chemotherapies in addition to surgical intervention have been evaluated over the last decades, the 5-year survival rate of pancreatic cancer patients is still only 7 % [1]. Recently, clinical studies demonstrated prolonged survival of patients after the application of novel combinatorial chemotherapies such as FOLFIRINOX or the combination of gemcitabine with nab-paclitaxel or S-1 [2–4]. However, these novel therapies can prolong the survival of patients only by a few weeks. Since progress in the treatment of pancreatic ductal adenocarcinoma (PDA) has been modest, the evaluation of novel therapies and the investigation of all pathophysiological aspects of PDA continue to be essential.

The evaluation of therapies in tissue culture along with the analysis in an orthotopic cancer model combines the advantages of in vitro studies with the advantages of a clinically relevant animal model [5, 6]. Tissue culture allows the evaluation of combinatorial therapies on isolated cancer cells in a fast and cost efficient way, whereas a syngeneic orthotopic cancer model allows the in vivo evaluation of therapies while considering clinically relevant factors such as a desmoplastic reaction, an intact immune system, and pharmacokinetic aspects of applied therapies [7]. It has been suggested that mice with orthotopically implanted syngeneic tumors are clinically more relevant than alternative animal models [7].

However, only few cell lines are available for PDA in a syngeneic orthotopic cancer model. Panc02 cells, derived from C57BL/6 mice after treating the animals with 3-methyl-cholanthrene, were originally characterized more than 30 years ago [8]. These pancreatic adenocarcinoma cells have been used in many studies but have probably accumulated genetic changes over time in distinct laboratories. Recently, the need for additional carcinoma cell lines has been addressed by characterizing cancer cells, which were isolated from genetically modified mice with a C57BL/6 or mixed B6/129 background [9, 10]. However, cells derived from a mixed background require the evaluation of histocompatibility of recipient mice [9]. The 6606PDA cell line, which is syngeneic to the C57BL/6J mouse strain, has first been described in 2011 [11]. This cell line has been reported to generate more differentiated glandular adenocarcinomas than Panc02 cells when injected into the pancreas head [12].

The purpose of this study was to compare the characteristics of two novel cell lines, 7265PDA and 66061, with 6606PDA cells in vitro. In addition, we evaluated these cell lines in a mouse model after orthotopic injection by analyzing tumor growth using magnetic resonance imaging (MRI) at two different time points.

## Results and discussion

### Characterization of 6606PDA, 6606l, and 7265PDA cells in vitro

**Fig. 1:**
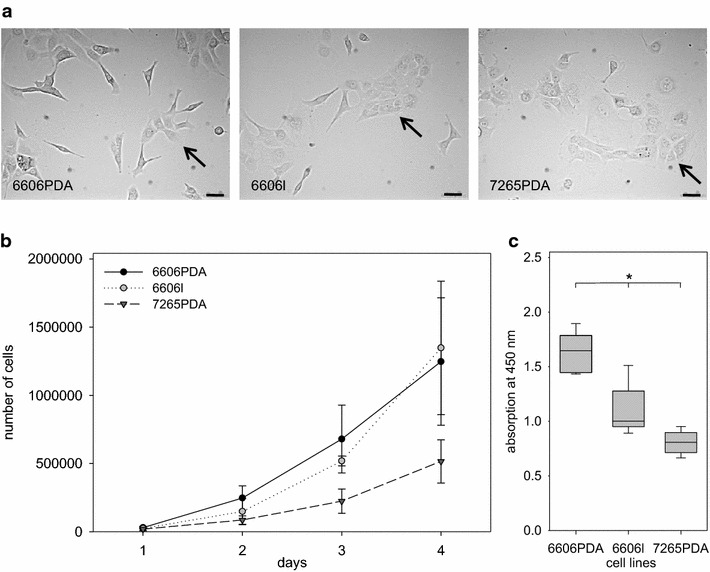
Characterization of carcinoma cell lines in tissue culture. **a** Characteristic images of 6606PDA, 6606l, and 7265PDA cells grown on tissue culture plates with partial epithelioid morphology (*arrow*). **b** Quantification of cell expansion of indicated cells lines by direct counting of cells (number of independent experiments: 4 for each cell line). **c** Quantification of cell expansion of indicated cells lines by WST-1 assay. Significant differences (*P ≤ 0.009) between all three cell lines are indicated in the *box plot* (number of independent experiments: 6 for each cell line). *Bar* 50 μm

When plated on tissue culture dishes, the cells of the murine cell lines, 6606PDA and 6606l, showed a similar morphology. At low cell density these cells had a stretched fibroblast like appearance (Fig. 1a). However, when these cells could form intercellular contacts, their appearance changed to a distinct flat epithelioid morphology (Fig. 1a). 7265 PDA cells preferentially showed a flat epithelioid morphology (Fig. 1a). Proliferation of these cells was quantified directly by counting them or indirectly by WST-Assay (Fig. 1b, c). When the cells were subconfluent, 6606PDA cells proliferated faster than 6606l cells, whereas 7265PDA cells were the slowest to divide (Fig. 1b, c).

**Fig. 2:**
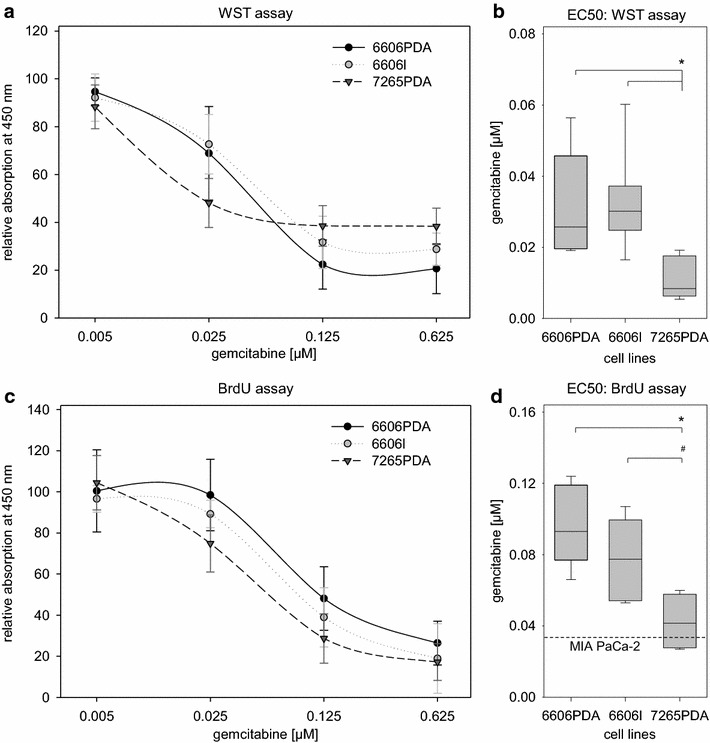
Inhibition of proliferation by gemcitabine. **a** Quantification of cell proliferation of 6606PDA, 6606l, and 7265PDA cells grown in media containing the indicated gemcitabine concentrations using WST-1 assays. **b** Comparison of EC50 values for each indicated cell line as measured by WST-1 assay. **c** Quantification of gemcitabine dependent cell proliferation of 6606PDA, 6606l, and 7265PDA cells using BrdU incorporation assays. **d** Presentation of EC50 values for each indicated cell line as measured by BrdU incorporation and comparison to published EC50 values from MIA PaCa-2 cells [13]. Significant differences (*P = 0.001) and a tendentious difference (^#^P = 0.026) are shown in the *box plots* (number of independent experiments: n = 7 for 6606PDA; n = 8 for 6606l, n = 7 for 7265PDA in **a** and **b**; n = 7 for 6606PDA; n = 6 for 6606l, n = 6 in **c** and **d**)

**Fig. 3:**
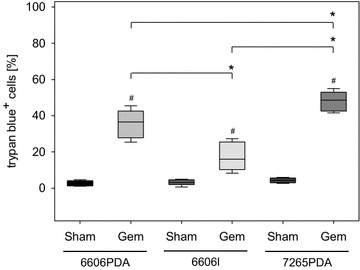
Induction of cell death by gemcitabine. **a** Quantification of cell death of 6606PDA, 6606l, and 7265PDA cells grown in unsupplemented medium (Sham) or medium supplemented with 625 nM gemcitabine (Gem) using a trypan blue exclusion assay. Significant differences between indicated cohorts (*P ≤ 0.005); and significant differences between cells of the identical cell line (^#^P ≤ 0.001) grown in gemcitabine versus control medium are shown in the *box plot* (number of independent experiments: n = 10 for 6606PDA; n = 8 for 6606l, n = 7 for 7265PDA)

In order to evaluate if these cell lines are sensitive to gemcitabine, an established drug for chemoterapy, we treated all three cell lines with distinct concentrations of gemcitabine and quantified cell proliferation indirectly by WST-assay (Fig. 2a, b) or directly by measuring 5-bromo-2′-deoxyuridine (BrdU) incorporation (Fig. 2c, d). A concentration dependent inhibition of proliferation was observed with all three cell lines using either assay (Fig. 2a, c). For 7265PDA cells, however, a lower half maximal effective concentration (EC50) of gemcitabine could be defined compared to those of 6606PDA or 6606l cells (Fig. 2b, d). All three cell lines exhibited EC50 values for gemcitabine similar to human cell lines such as MIA PaCa-2 cells [13]. We also quantified cell death of these cell lines after treatment with gemcitabine. Gemcitabine strongly induced cell death in all three cell lines (Fig. 3). Gemcitabine induced a higher percentage of dead cells in 7265PDA cells when compared to gemcitabine treated 6606PDA and 6606l cells (Fig. 3). This confirmed that 7265PDA cells are more sensitive to gemcitabine (Fig. 3). A lower percentage of dead cells was observed after gemcitabine treatment of 6606l cells when compared to 6606PDA cells, although inhibition of proliferation after gemcitabine was similar between 6606PDA and 6606l cells (Figs. 2, 3). Probably mutations, which limit cell death in response to gemcitabine, accumulated in the 6606l cell line. Due to the observed sensitivity to gemcitabine all three cell lines should be especially useful in evaluating additional chemotherapeutical agents in combination with gemcitabine in future. Such preclinical studies have been published for several other cell lines such as AsPC-1, SUIT-2, MIA PaCa-2, or Panc02 cells [14–17].

### Characterization of 6606PDA, 6606l, and 7265PDA cells in vivo

**Fig. 4:**
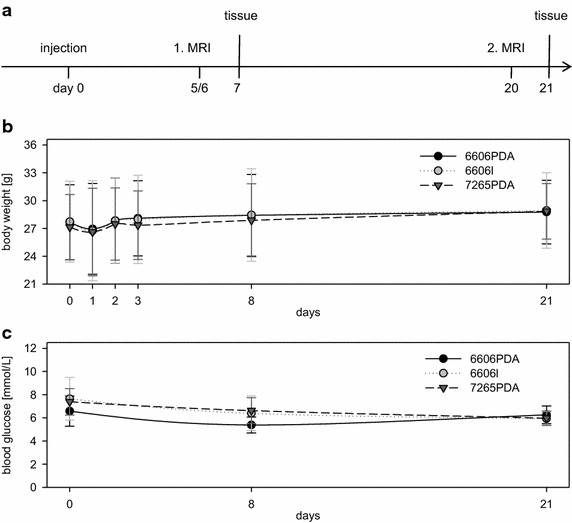
The Murine carcinoma model. **a** Experimental protocol: injection of carcinoma cells into the pancreas head on day 0, first MRI during early phase (day 5 or 6), second MRI during the late phase of tumor growth (day 20), and analysis of the tissue on day 7 or 21. **b** Mouse body weight at indicated time points after injection of 6606PDA, 6606l, or 7265PDA cells. **c** Blood glucose concentration at indicated time points after injection of 6606PDA, 6606l, or 7265PDA cells. Number of animals analyzed: n = 6 for 6606PDA; n = 6 for 6606l, n = 11 for 7265PDA; 2 independent experiments

In order to evaluate, if these cell lines can be used in a syngeneic orthotopic pancreas carcinoma model, these cells were injected into the pancreas head of C57BL/6J mice on day 0 and the pancreata were analyzed during the early phase (on day 5–7) and during the late phase, on day 20 or 21 (Fig. 4a). After injection of either cell line an insignificant postoperative decline in body weight was observed, but no cachexia developed within 3 weeks (Fig. 4b). The blood glucose concentration was also stable throughout this study (Fig. 4c). These data suggest that within 3 weeks these cell lines cannot be used for examining cachexia or diabetes as paraneoplastic phenomena that have been suggested to occur in patients with pancreatic cancer [18, 19].

**Fig. 5:**
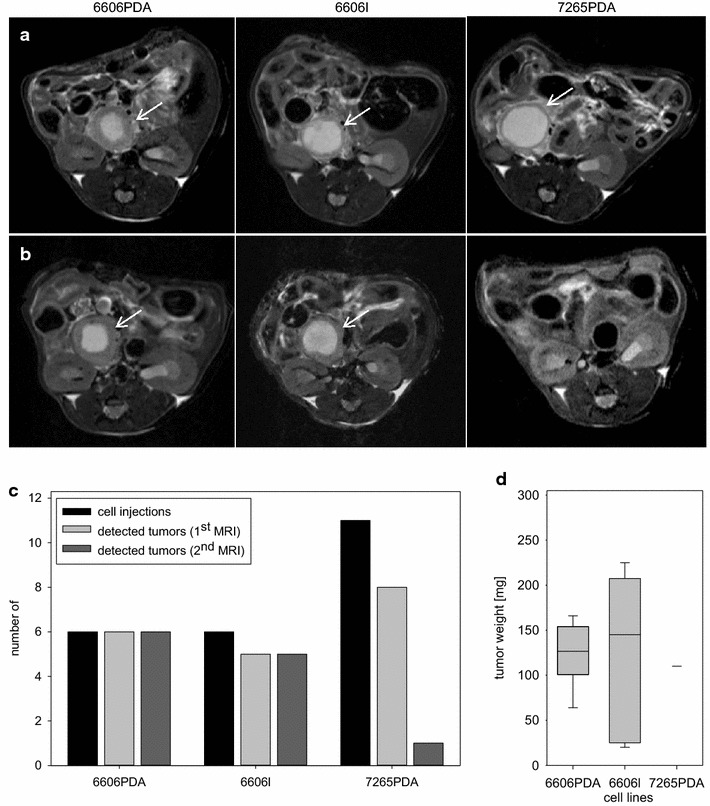
Characterization of carcinoma cell lines in vivo. **a** Characteristic T2-weighted MRI of 6606PDA, 6606l, and 7265PDA cell derived carcinomas (*arrows*) during early phase of tumor growth (day 5/6). **b** Characteristic T2-weighted MRI of carcinomas (*arrows*) during late phase of tumor growth (day 20). **c** Graphical presentation of the number of cell injections carried out and the number of detected tumors at the first MRI or the second MRI for 6606PDA, 6606l, and 7265PDA cells (number of mice analyzed: n = 6 for 6606PDA; n = 6 for 6606l, n = 11 for 7265PDA). **d** Comparison of tumor weight for indicated cell lines on day 21. Number of tumors analyzed: n = 6 for 6606PDA; n = 5 for 6606l, n = 1 for 7265PDA; 2 independent experiments

To monitor tumors, we assessed the pancreata of mice by 7 T MRI during the early phase (day 5 or 6) and during the late phase of tumor growth (day 20). Carcinomas could be identified during the early phase irrespective of the cell line used (Fig. 5a). Carcinomas could also be identified easily during the late phase when 6606PDA and 6606l cells were injected. However, in most cases carcinomas could not be identified when 7265PDA cells were injected (Fig. 5b). We quantified this observation and noticed that after injecting a total number of 6 animals with 6606PDA cells 6 carcinomas could be identified during the early phase and 6 carcinomas during the late phase of tumor growth (Fig. 5c). When injecting a total number of 6 mice with 6606l cells 5 carcinomas could be identified during the early phase and 4 carcinomas during the late phase (Fig. 5c). The median tumor weight of 6606PDA and 6606l derived tumors on day 21 was 126 mg (interquartile range 101–154 mg) and 145 mg (interquartile range 25–208 mg), respectively (Fig. 5d). Thus, these two cell lines generate carcinomas in immunocompetent C57BL/6J mice and should be particularly valuable in studying various aspects of cancer immunology. When injecting a total number of 11 mice with 7265PDA cells 8 carcinomas could be identified during the early phase of tumor growth and only 1 carcinoma could be observed during the late phase with a tumor weight of 110 mg (Fig. 5c, d). Thus, seven mice have completely lost the tumor within 2 weeks. In this group the number of animals was increased to 11 mice in order to verify the reproducibility of the observed carcinoma remission. Macroscopic inspection of the pancreas after laparotomy of the animals verified that MRI accurately identified the presence or the absence of carcinomas. We also injected a total number of four immunocompromised NMRI-Foxn1^nu/nu^ mice with 7265PDA cells and observed that all mice developed carcinomas within 21 days (median tumor weight: 325 mg; interquartile range 216–563 mg). The remission of carcinomas after injection of 7265PDA cells in C57BL/6J mice suggests that this mouse strain is not syngeneic to this cell line. Indeed, information obtained from the laboratory of David Tuveson (Cambridge, UK) suggests that this cell line was isolated from a mouse with a mixed Bl6/129 background. Thus, for 7265PDA cells the use of immunocompromised mouse strains or mice with matched histocompatibility will be mandatory.

**Fig. 6:**
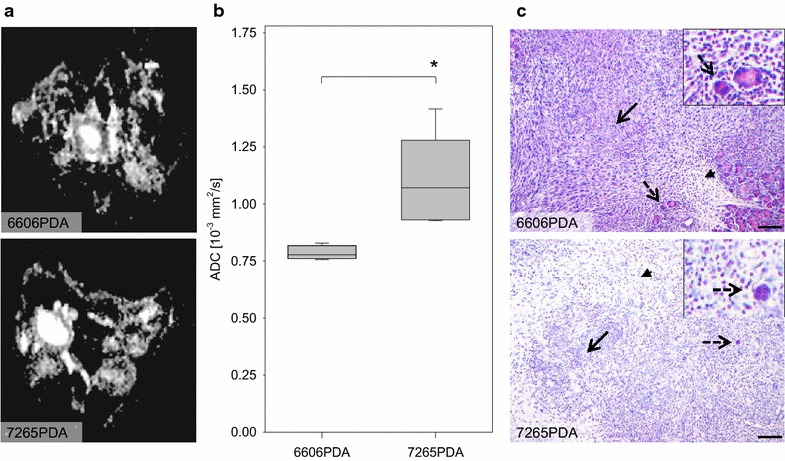
Characterization of carcinomas by diffusion-weighted MRI and histology during the early phase of tumor growth. **a** Characteristic diffusion-weighted MRI of 6606PDA and 7265PDA cell derived carcinomas (day 5/6). **b** Quantification of water diffusion of 6606PDA and 7265PDA cell derived carcinomas by diffusion-weighted MRI, as given by the ADC value. **c** characteristic haematoxylin/eosin (H/E) staining of histological sections of carcinomas during the early phase of tumor growth (*dotted arrow* pancreatic acinar cells, *arrow* cancer cells, *arrowhead* oedema with infiltrating inflammatory cells). A significant difference between indicated cohorts (*P = 0.01) is shown in the *box plot* (number of tumors analyzed: n = 4 for 6606PDA, n = 6 for 7265PDA; 2 independent experiments). *Bar* 100 μm

To characterize differences between carcinomas derived from 6606PDA cells and 7265PDA diffusion-weighted MRI was performed during the early phase of tumor growth (Fig. 6a). Interestingly the calculated apparent diffusion coefficient (ADC), measured at the edge of both classes of carcinomas, was higher in carcinomas derived from 7265PDA cells than in carcinomas derived from 6606PDA cells (Fig. 6b). This observation correlated well with the histology of these tumors. 6606PDA cell derived carcinomas were characterized by many densely packed cancer cells (Fig. 6c upper panel). Inflammatory cells surrounded these carcinomas, but only few inflammatory cells were observed within these carcinomas (Fig. 6c upper panel). 7265PDA cell derived carcinomas were characterized by oedema, massive infiltration of inflammatory cells, and few loosely packed cancer cells (Fig. 6c lower panel). These data suggest that less than a week after injection of 7265PDA cells the immune system of C57BL/6J mice prevented the accumulation of densely packed cancer cells. Instead, infiltrating inflammatory cells lead to a local oedema which results in a higher ADC value when diffusion-weighted MRI was performed.

**Fig. 7:**
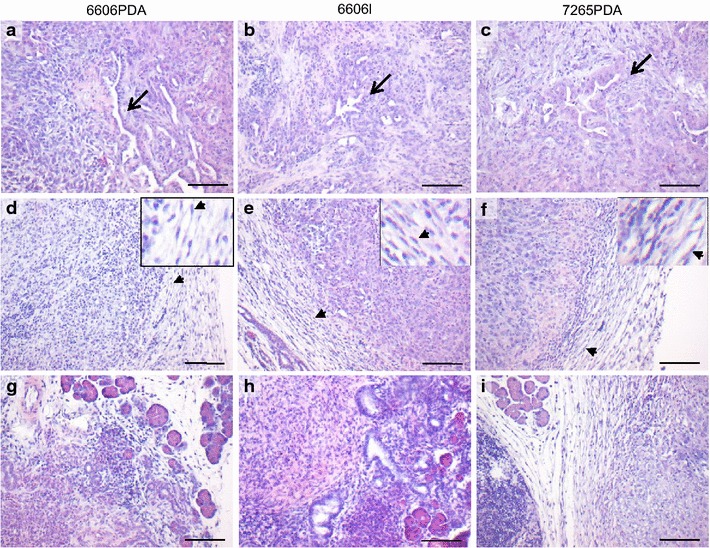
Characterization of carcinomas by H/E staining during the late phase of tumor growth. **a**–**c** Histological images of carcinomas, which were derived from 6606PDA, 6606l, or 7265PDA cells, show cells with epithelial morphology on day 21 (*arrows*). **d**–**f** Representative histological images of carcinomas, which were derived from the indicated cell lines, show that the carcinomas are mostly encapsulated by fibroblast like cells (*arrowheads*). **g**–**i** In few locations lack of encapsulation was observed in 6606PDA (**g**) or 6606l (**h**) but not in 7265PDA (**i**) derived carcinomas (selected histological images). Number of tumors analyzed: n = 6 for 6606PDA; n = 5 for 6606l, n = 1 for 7265PDA (2 independent experiments). *Bar* 100 μm

On day 21 cells with epithelial morphology could be observed in carcinomas derived from all three cell lines (Fig. 7a–c). All carcinomas were largely encapsulated by fibroblast like cells (Fig. 7d–f). On individual histological sections of 6606PDA or 6606l derived tumors, the carcinomas lacked complete encapsulation by fibroblast like cells (Fig. 7g–h). This suggests moderate infiltrative growth of the cancer cells (Fig. 7g–h). A deficiency in encapsulation was not observed in the one carcinoma derived from 7265PDA cells (Fig. 7i). Consistent with the observation that infiltrative growth was hardly observed on day 21, we did also not detect any hepatic metastasis by macroscopic evaluation of the liver (data not shown). Since during the early phase (day 5 or day 6) the carcinomas derived from these three cell lines were barely encapsulated by fibroblast like cells (Fig. 6c, d, and data not shown), the development of desmoplasia could be studied in this animal model between day 6 and day 21.

## Conclusion

The presented experiments describe the applicability of the carcinoma cell lines 6606PDA, 7265PDA, and 6606l for in vitro as well as in vivo studies. The data suggest that (1) all three cell lines will be useful in studying novel combinatorial therapies in vitro using gemcitabine, (2) the 6606PDA and 6606l cells might be very suitable in studying various aspects of cancer immunology in C57BL/6J mice, and that (3) diffusion weighted MRI can be used to define immune mediated cancer rejection.

## Methods

### Cell culture

The murine cell lines, 6606PDA and 6606l, were isolated from pancreatic carcinoma or a liver metastasis detected in a mouse after p48-cre induced expression of the KRAS^G12D^ oncogene in the pancreas [20]. The 7265PDA cells were derived from a pancreatic carcinoma, which developed after pdx1-creER induced expression of p53^R172H^ and KRAS^G12D^. These three cell lines were a generous gift from Prof. Tuveson (Cambridge, UK) and were isolated in his lab according to Schreiber et al. [21]. Briefly, the tumors were dissected, minced, and digested at 37 °C in a Hank’s balanced salt solution containing 2 mg/mL type V collagenase (Sigma, St. Louis, MO, USA). The material was then filtered through nylon mesh and the protease was inactivated by the addition of Dulbecco’s modified Eagle medium/F12 (Invitrogen, Carlsbad, CA, USA) supplemented with 10 % fetal bovine serum. For all assays the cells were grown in DMEM high glucose medium (Biochrom GmbH, Berlin, Germany) with 10 % fetal calf serum on uncoated plastic dishes. Expansion of all three cells lines was quantified directly by plating 4 × 10^3^ cells per well in a 12 well plate and counting cells after 24, 48, 72, or 96 h. Expansion of cells was also determined indirectly by WST-assay after plating of 4 × 10^3^ cells per well in a 96 well plate, growing the cells 48 h, and determining cell metabolism with the Cell Proliferation Reagent WST-1 (Roche Diagnostics, Mannheim, Germany). To evaluate inhibition of proliferation by gemcitabine 4 × 10^3^ cells per well were plated in a 96 well plate, grown for 24 h, and treated with the indicated concentration of gemcitabine and the BrdU labeling reagent (Roche Diagnostics, Mannheim, Germany) for additional 24 h. The incorporation of BrdU was then quantified with the colorimetric Cell Proliferation ELISA (Roche Diagnostics). To evaluate induction of cell death by gemcitabine 3 × 10^4^ cells per well were plated in a 24 well plate, incubated for 24 h, and treated with 625 nM gemcitabine or medium lacking gemcitabine for additional 48 h. Cell death was then quantified by trypan blue staining solution (Life Technologies, CA, USA).

### Animals and the syngeneic orthotopic carcinoma model

Male C57BL/6J and NMRI-Foxn1^nu/nu^ mice were purchased from The Jackson Laboratory (Bar Harbor, ME) and bred in our local animal facility. Mice were kept on water and standard laboratory chow ad libitum and all experiments were executed in accordance with German legislation and the EU-directive 2010/63/EU. As published previously, laparotomy was performed on anesthetized mice (1.2–2.5 % isoflurane), 2.5 × 10^5^ carcinoma cells were injected (single injection per animal) into the pancreas head, and the abdominal cavity was closed by sutures [22]. For pain relief 5 mg/kg carprofen (Pfizer GmbH, Berlin, Germany) was injected (sc) before surgery and 800 mg/L metamizol (Ratiopharm GmbH, Ulm, Germany) was added to the drinking water until euthanasia of the mice. For sampling blood and tissues animals were anesthetized with 90 mg/kg ketamine (bela-pharm, Vechta, Germany) and 7 mg/kg xylazine (Bayer Health Care, Leverkusen, Germany).

### Analysis of the blood and the tissue

Blood glucose concentrations were measured with the blood glucose meter Contour (Bayer Vital, Leverkusen, Germany) and the tissue was sampled on days defined in Fig. 4a. The tissue was processed as described previously [23]. The histology of carcinomas was evaluated on haematoxylin and eosin (H/E) stained paraffin tissue sections.

### Morphological and diffusion-weighted 7 T MRI

MRI was performed on anesthetized (1.2–2.5 % isoflurane) mice at day 5–6 and day 20. Animals were scanned in 7 T small animal MRI (ClinScan, 7.0 T, 290 mT/m gradient strength) with a whole mouse body coil (Bruker, Ettlingen, Germany). The imaging protocol included morphological T2 weighted turbo spin echo (T2w-TSE) and diffusion weighted imaging (DWI). Tumor sizes were assessed in high resolution T2-weighted images of transversal plane images (TR: due to respiratory gating approx. 1900 ms; TE: 45.0 ms; FA: 180°; FoV: 40 mm × 40 mm; matrix: 240 × 320; voxel size: 0.16 × 0.125 × 0.7 mm^3^, 24 slices, acquisition time: 9:46 min).

The ADC value was calculated from echo planar imaging DWI-sequence (two b values, b_1_ = 0, b_2_ = 800) TR: 9000 ms; TE: 85 ms; FA: 90°; FoV: 35 mm × 35 mm; matrix: 256 × 256; 10 slices of 0.7 mm per slice.

### Analysis of MRI data

Images were analyzed employing Slicer3D (National Institutes of Health, Bethesda, Maryland, USA) and ImageJ (National Institutes of Health, Bethesda, Maryland, USA) for ROI placement during ADC evaluation. For evaluation of necrosis and vital tumor tissue T2w images and ADC-maps were superimposed. For each carcinoma the mean ADC of three ROIs, fitted to the outer tumor tissue rim, was calculated.

### Statistics

Data presentation and statistics were performed as described previously [22, 23]. The significance of differences was evaluated using a Mann–Whitney rank-sum test followed by the correction for the accumulation of the α error by considering the number of meaningful comparisons. Differences with P ≤ 0.05, divided by the number of meaningful comparisons, were considered to be significant.

## Abbreviations

ADC: Apparent diffusion coefficient
BrdU: 5-Bromo-2′-deoxyuridine
DWI: Diffusion weighted imaging
EC50: Half maximal effective concentration
MRI: Magnetic resonance imaging
H/E: Haematoxylin/eosin
